# Assessing causal estimates of the association of obesity-related traits with coronary artery disease using a Mendelian randomization approach

**DOI:** 10.1038/s41598-018-25305-y

**Published:** 2018-05-08

**Authors:** Xue Zhang, Wan-Qiang Lv, Bo Qiu, Li-Jun Zhang, Jian Qin, Feng-Juan Tang, Hai-Tao Wang, Hua-Jie Li, Ya-Rong Hao

**Affiliations:** 1 Department of Geriatrics, Renmin Hospital of Wuhan University, Wuhan, 430060 Hubei Province P. R. China; 2 Department of Epidemiology, College of Public Health, Zhengzhou University, Zhengzhou, NO.100 Kexue Road, High-Tech Development Zone Of States, Zhengzhou, P. R. China; 3 Department of Orthopaedics, Renmin Hospital of Wuhan University, Wuhan, 430060 Hubei Province P. R. China; 4 Central Laboratory, Renmin Hospital of Wuhan University, Wuhan, 430060 Hubei Province P. R. China

## Abstract

Obesity-related traits have been associated with coronary artery disease (CAD) in observational studies, but these associations may be biased by confounding factors and reverse causation. In this study, we specifically conducted two-sample Mendelian randomization (MR) analyses to overcome these limitations and test the associations of obesity-related traits (other than body mass index (BMI)) (n = 322,154) with CAD (22,233 cases and 64,762 controls) by using summary-level data from previous studies. The methods utilized to estimate these associations included the inverse-variance weighted method, the weighted median method and MR-Egger regression. Our results supported causal effects of BMI, hip circumference (HC), waist circumference (WC), and waist-hip ratio (WHR) on CAD. The associations of BMI-adjusted HC and WC with CAD were reversed, unlike that of WHR. In MR analyses excluding overlapping single nucleotide polymorphisms (SNPs) from obesity-related traits, the associations of these traits with CAD were preserved. The associations of BMI-adjusted HC and WC with CAD require further investigation, as collider stratification may be occurring. Additionally, central adiposity (measured by WHR) separated from general adiposity (measured by BMI) and general adiposity might pose similar risks for CAD. In clinical practice, physicians should pay attention to the potential effects of different obesity-related traits on CAD.

## Introduction

Epidemiological studies have estimated that the prevalence of overweight/obesity increased by approximately 41% between 1980 and 2013^1^. Overweight/obesity was associated with an increased prevalence of coronary heart disease (CAD) in most observational studies^2,3^, making it a major contributor or risk factor to the rise in CAD^4^. Moreover, CAD has been one of the leading causes of morbidity and mortality worldwide^1^.

Observational studies have shown that general adiposity is an independent predictor of CAD^5^. A previous study demonstrated that every 1 kg/m^2^ increase in body mass index (BMI) leads to a 5–7% increase in the incidence of CAD across all BMI categories^6^, supporting a positive association between high BMI and risk of CAD. Similarly, a large meta-analysis showed that obese participants had a significantly greater risk of CAD (relative risk - RR 1.81, 95% confidence interval - CI 1.56–2.10) after adjusting for age, sex, physical activities, and smoking^7^. The accumulation of body fat has been shown to lead to classic metabolic abnormalities, particularly insulin resistance^8^. Insulin resistance may increase cardiovascular risk through increased activity of the systemic renin-angiotensin system^9^, subclinical inflammation (estimated by C-reactive protein)^10^, and lower natriuretic peptide levels^11^, all of which combine to further increase the likelihood of developing CAD^12^. Inconsistent findings from these observational studies reflect the limitations of observational studies, which cannot fully eliminate the influence of confounding factors and are susceptible to recall bias and reverse causation^13^.

Studies have reported that the impact of adiposity on the risk of CAD is determined by the degree to which fat accumulates as well as its location, but BMI has been widely used in research^14^. BMI may overestimate body fat in people who are very muscular and underestimate body fat in those who have lost muscle mass^15^. One study reported similar associations of general adiposity (measured by BMI, over 30 kg/m^2^) and central adiposity (measured by waist-hip ratio (WHR), above 0.90 for males and above 0.85 for females) with CAD^16^, but other studies indicated that central obesity (measured by either waist circumference (WC) or WHR) may have a stronger association with CAD^17,18^.

Mendelian randomization (MR) is a technique that uses allelic variants as instrumental variables (IVs) to assess whether the associations between risk factors and disease are causal^19,20^. As variants are randomly distributed across the population and are robustly associated with risk factor of interest, taking advantage of IVs as proxies for risk factor can avoid confounding bias from environmental factors and eliminate potential reverse causality. Compared to traditional observational studies, MR provides a better option for determining whether associations are causal. Although some MR studies have been applied to assess the association of adiposity with CAD^21–24^, these have mainly focused on BMI as an exposure proxy solely for adiposity and used small sample sizes. MR estimates from a two-sample analysis (in which data on the risk factor and outcome are derived from non-overlapping genome-wide association study (GWAS) datasets) are less biased, and any bias is in the direction of the null. In this study, two-sample MR analyses were performed to quantify and contrast causal estimates of the association of different obesity-related traits with CAD, including BMI, hip circumference (HC), WC, and WHR both with and without adjustment for BMI. We hypothesized that this method could uncover causal relationships between obesity-related traits and CAD and extend the measures of adiposity and fat distribution as exposure proxies.

## Results

### MR analysis of BMI and CAD

**Figure 1:**
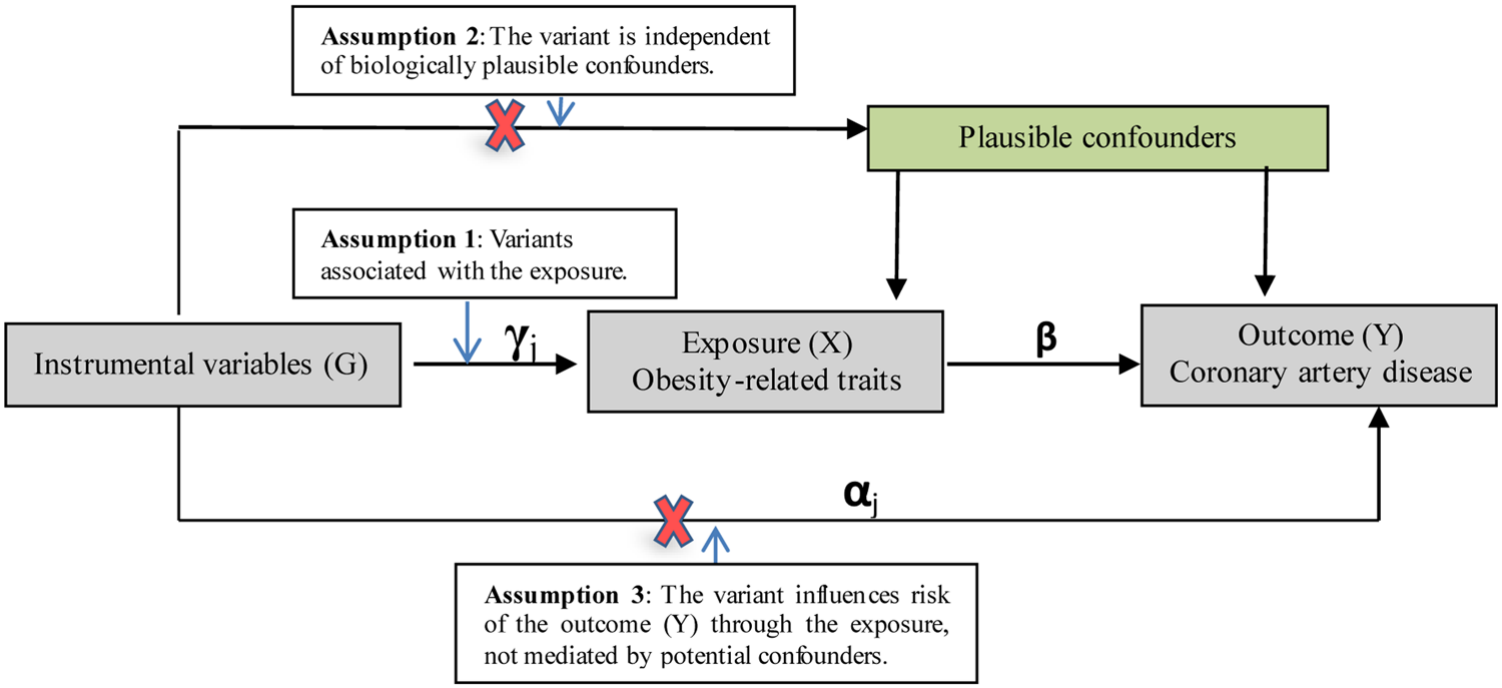
Mendelian randomization model and three key assumptions of a Mendelian randomization analysis. The coefficient γ represents the association of the variant with the exposure, and α represents the association of the variant with the outcome that is not mediated by the exposure and other potential confounders. The coefficient β represents the causal effect of the exposure on the outcome.

At the standard threshold of genome-wide significance (p < 5 × 10^−8^), 68 single nucleotide polymorphisms (SNPs) were identified for BMI (linkage disequilibrium (LD) *r*^2^ < 0.001) (Supplemental Table S1), accounting for 1.9% of the variance in BMI in the summarized data, thus validating assumption 1 in Fig. 1. We found a positive association of BMI with CAD from the IVW results (odds ratio (OR) 1.37, 95% CI 1.15–1.63). The intercept from the MR-Egger regression was −0.001 (95%CI −0.015–0.012; p = 0.834), indicating that the association was not biased by any unbalanced horizontal pleiotropy. The causal estimate from MR-Egger was 1.44 (95% CI 0.86–2.42), and the estimated effect size from the weighted median method was consistent with the IVW results (OR 1.37, 95% CI 1.10–1.71) (Table 1). Taking into account the heterogeneity in the analysis (p = 0.06), we used the forest and funnel plot (Supplemental Figs 1 and 2). The plots indicated that three SNPs might add heterogeneity, including rs758747, rs205262 and rs17724992. After excluding these SNPs, we showed that the association of BMI and CAD was preserved (IVW-OR 1.33, 95% CI 1.14–1.55; Weighted median-OR 1.37, 95% CI 1.14–1.55; 1.09–1.72; heterogeneity p = 0.34). The results of MR leave-one-out analysis suggested that the estimated association of BMI with CAD is not disproportionately influenced by a single SNP (Supplemental Fig. 3).

**Table 1:** Causal estimates of the association of obesity-related traits on risk of CAD.

Methods	Exposure						
	BMI	HC	HC*	WC	WC*	WHR	WHR*
Total sample size (N)	322,154	210,088	210,088	210,088	210,088	210,088	210,088
Numbers of IVs	68	52	73	41	63	28	36
P_-heterogeneity_	0.06	5.91E-04	0.025	9.09E-04	4.77E-05	0.222	0.028
R^2^	0.019	0.018	0.026	0.012	0.017	0.007	0.011
**IVW method**							
OR	1.37	1.10	0.83	1.39	0.90	1.46	1.42
95% CI	1.15–1.63	0.89–1.36	0.71–0.96	1.06–1.84	0.72–1.13	1.17–1.91	1.12–1.81
p-value	4.74E-04	0.385	0.012	0.018	0.371	0.006	0.004
**MR-Egger test**							
OR_-slope_	1.44	1.53	1.03	1.87	1.25	3.47	0.93
95% CI_-slope_	0.86–2.42	0.83–2.85	0.580–1.811	0.70–4.94	0.455–3.415	1.05–11.58	0.29–3.02
p-value_-slope_	0.169	0.182	0.932	0.217	0.670	0.052	0.901
intercept	−0.001	−0.011	−0.007	−0.008	−0.009	−0.023	0.012
95% CI	−0.015–0.012	−0.029–0.008	−0.024–0.010	−0.034–0.017	−0.034–0.017	−0.053–0.008	−0.021–0.046
p-value_-intercept_	0.834	0.266	0.440	0.544	0.518	0.159	0.474
**Weighted median**							
OR	1.37	1.44	0.85	1.55	1.08	1.55	1.25
95% CI	1.10–1.71	1.12–1.84	0.70–0.97	1.16–2.07	0.83–1.40	1.08–2.20	1.07–1.66
p-value	0.005	0.005	0.016	0.003	0.540	0.017	0.014

*Indicates that this trait was adjusted for body mass index (BMI) in our study. R^2^ refers to the proportion of variance of the exposure explained by the instruments. Hip circumference (HC), waist circumference (WC), waist-hip ratio (WHR), odds ratio (OR), confidence interval (CI), instrumental variable (IV), inverse variance weighted (IVW). In the table, the standard deviations (SDs) for BMI, HC, WC and WHR are 4.770 (kg/m^2^), 8.455 (cm), 12.520 (cm) and 0.074, respectively. OR means that the change in odds in CAD with a 1-SD increase in each obesity-related trait. P-heterogeneity is derived from the Q statistic, and p-intercept is derived from the MR-Egger test.

### MR analysis of HC and CAD

With the same standard, 52 HC SNPs and 73 BMI-adjusted HC SNPs were identified (Supplemental Tables S2 and S3), accounting for 1.8% and 2.6% of the variance, respectively. In the IV analysis of HC with CAD, we found a positive association of HC with CAD from the IVW results (OR 1.10, 95% CI 0.89–1.36). The intercept from the MR-Egger test did not reveal any unbalanced horizontal pleiotropy (intercept p-value = 0.27). The causal estimate from MR-Egger was 1.53 (95% CI 0.83–2.85). However, the heterogeneity test supported heterogeneity in the data (p = 5.9 × 10^−4^). As suggested in the study by Jack Bowden and coworkers^25^, we conducted MR analysis using the weighted median method to take into account this heterogeneity, and the OR for CAD was 1.44 (95% CI 1.12–1.84) with HC. MR leave-one-out analysis suggested that the estimated association is not disproportionately influenced by a single SNP (Supplemental Fig. 4). Our results provided evidence that the risk of CAD decreased with increased BMI-adjusted HC from the IVW results (OR 0.83, 95% CI 0.71–0.96). From the MR-Egger test, the intercept did not reveal any unbalanced horizontal pleiotropy (intercept p = 0.440), and we found a positive but imprecise association of BMI-adjusted HC with CAD (OR 1.03, 95% CI 0.58–1.81). Taking into account the heterogeneity in the IVs (p = 0.025), the result from the weighted median method was consistent with the IVW result (OR 0.85, 95% CI 0.70–0.97). MR leave-one-out analysis suggested that the estimated association is not disproportionately influenced by a single SNP **(**Supplemental Fig. 5**)**.

### MR analysis of WC and CAD

We identified 41 WC SNPs and 63 BMI-adjusted WC SNPs (Supplemental Tables S4 and S5), accounting for 1.2% and 1.7% of the variance, respectively. In the IV analysis of the effect of WC on the risk of CAD, we found a positive association of WC with CAD from the IVW results (OR 1.39, 95% CI 1.06–1.84). From the MR-Egger test, the intercept did not reveal any unbalanced horizontal pleiotropy (intercept p-value = 0.544), and the OR for CAD was 1.87 (95% CI 0.70–4.94). Taking into account the heterogeneity in the analysis (p = 9.1 × 10^−4^), the result from the weighted median was consistent with the IVW results (OR 1.55, 95% CI 1.16–2.07). MR leave-one-out analysis suggested that the estimated association is not disproportionately influenced by a single SNP (Supplemental Fig. 6). The IVW results indicated that the risk of CAD decreased with increased BMI-adjusted WC (OR 0.90, 95% CI 0.72–1.13). From the MR-Egger test, the intercept did not reveal horizontal pleiotropy (p-value = 0.518), and the causal estimate for CAD was 1.25 (95% CI 0.46–3.42). When taking into account the heterogeneity (p = 4.8 × 10^−5^), we found a positive association of BMI-adjusted WC with the risk of CAD (OR 1.08, 95% CI 0.83–1.40). MR leave-one-out analysis suggested that the estimated association is not disproportionately influenced by a single SNP (Supplemental Fig. 7).

### MR analysis of WHR and CAD

We identified 28 WHR SNPs and 36 BMI-adjusted WHR SNPs (Supplemental Tables S6 and S7), accounting for 0.7% and 1.1% of the variance, respectively. In the IV analysis of the effect of WHR on the risk of CAD, we found a positive association of WHR with CAD from the IVW results (OR 1.46, 95% CI 1.17–1.91). From the MR-Egger test, the intercept did not reveal any unbalanced horizontal pleiotropy (p-value = 0.159), and the OR for CAD was 3.47 (95% CI 1.05–11.58). The weighted median results were in line with the IVW results (OR 1.55, 95% CI 1.08–2.20). The heterogeneity test did not support heterogeneity in the data (p = 0.22). MR leave-one-out analysis suggested that the estimated association is not disproportionately influenced by a single SNP (Supplemental Fig. 8). The association of BMI-adjusted WHR with CAD was preserved, and the OR from the IVW results was 1.42 (95% CI 1.12–1.80), in line with the weighted median method (OR 1.25, 95% CI 1.07–1.66). From the MR-Egger test, the intercept did not reveal any unbalanced horizontal pleiotropy (p-value = 0.474), and the causal estimate for CAD was 0.93 (95% CI 0.29–3.02). MR leave-one-out analysis suggested that the estimated association is not disproportionately influenced by a single SNP (Supplemental Fig. 9).

### Overlapping SNPs between obesity-related traits

Detailed information on the overlap between the BMI, HC, WC, and WHR SNPs is shown in Supplementary Fig. 10. The overlapping SNPs between the BMI, BMI-adjusted HC, WC, and WHR SNPs are shown in Supplementary Fig. 11. To assess the specificity of the associations between obesity-related traits and CAD risk, we repeated the analyses after removing the overlapping SNPs. Overall, the associations of obesity-related traits with CAD were in line with the results obtained by ignoring the overlapping SNPs (Table 2).

**Table 2:** Causal estimates of the association of obesity-related traits on risk of CAD, excluding overlap SNPs.

Methods	Exposure						
	BMI	HC	HC*	WC	WC*	WHR	WHR*
**IVW method**							
OR	1.32	1.07	0.84	1.34	0.86	1.46	1.36
95% CI	1.06–1.66	0.82–1.39	0.71–0.98	0.89–2.01	0.65–1.12	1.11–1.94	1.03–1.80
p-value	0.015	0.628	0.033	0.163	0.260	0.007	0.030
**MR-Egger test**							
OR_-slope_	1.71	1.28	0.95	1.19	1.16	3.14	0.97
95% CI_-slope_	0.83–3.53	0.63–2.62	0.46–1.95	0.27–5.24	0.35–3.87	0.90–10.90	0.27–3.50
p-value_-slope_	0.152	0.506	0.893	0.822	0.813	0.084	0.965
intercept	−0.007	−0.006	−0.003	0.003	−0.008	−0.020	0.010
95% CI	−0.036	−0.028–0.016	−0.025–0.017	−0.035–0.041	−0.037–0.022	−0.052–0.012	−0.027–0.047
p-value_-intercept_	0.466	0.598	0.724	0.873	0.616	0.231	0.600
**Weighted median**							
OR	1.27	1.36	0.83	1.51	0.96	1.59	1.25
95% CI	1.04–1.68	1.07–1.84	0.67–0.96	1.03–2.29	0.74–1.32	1.10–2.29	0.87–1.78
p-value	0.024	0.035	0.040	0.041	0.930	0.011	0.222

*Indicates that this trait was adjusted for body mass index (BMI) in our study. R^2^ refers to the proportion of variance of the exposure explained by the instruments. Hip circumference (HC), waist circumference (WC), waist-hip ratio (WHR), odds ratio (OR), confidence interval (CI), instrumental variable (IV), inverse variance weighted (IVW). In the table, the standard deviations (SDs) for BMI, HC, WC and WHR are 4.770 (kg/m^2^), 8.455 (cm), 12.520 (cm) and 0.074, respectively. OR means that the change in odds in CAD with a 1-SD increase in each obesity-related trait. P-heterogeneity is derived from the Q statistic, and p-intercept is derived from the MR-Egger test.

## Discussion

In the present study, we conducted two-sample MR analyses to investigate whether the evidence supported the association of obesity-related traits, including BMI, HC, WC, and WHR, with CAD. Our study found evidence for positive associations of BMI, HC, WC, and WHR with the risk of CAD. The risk of CAD decreased with increased BMI-adjusted HC and WC, in contrast to the trend with WHR. However, further work is needed to investigate the association of HC and WC with CAD, especially after adjustment for BMI.

Some MR analyses have been applied to test the association of adiposity with CAD. However, these studies mainly focused on BMI as an exposure proxy solely for adiposity^22–24^, while our study extended other measures of adiposity and fat distribution as exposure proxies, including HC, WC and WHR, both adjusted and unadjusted for BMI. Moreover, these past studies included smaller sample sizes, leading to weaker IVs due to the limited numbers of SNPs identified as IVs for MR analyses^22–24^. Although several MR analyses have used larger sample sizes and larger numbers of SNPs as IVs^26,27^, our study had two main differences from previous studies. In those studies, the SNPs used as IVs were identified from a meta-analysis of GWASs of individuals from both European and non-European descent, but the datasets used for adiposity traits and CAD had different ancestors; this may be a potential source of bias. In contrast, our study only included the GWASs of individuals of European descent for both adiposity traits and CAD. Another difference is that we conducted two-sample MR analyses via the TwoSampleMR package, which can collate and harmonize summary-level data from publicly (and non-publicly) available GWAS datasets.

Our study provided evidence for positive associations of BMI and WHR with the risk of CAD, in line with most published epidemiological studies and MR studies^21–25^. Additionally, in MR analyses excluding overlapping SNPs from obesity-related traits, we still observed positive associations of these two traits with the risk of CAD. Our results indicated that a measure of central adiposity (measured by WHR) that is independent of general adiposity (measured by BMI) had a similar causal effect on CAD as general adiposity (p-values from the T-test > 0.05). This finding suggested the potential of MR approaches for investigating highly correlated adiposity measures that have previously proved challenging to disentangle in observational studies^28^. In clinical practice, physicians should pay attention to the potential effects of different obesity-related traits on the risk of CAD.

Studies have suggested that, for a given body size, larger HC is associated with a better cardiovascular risk profile after adjusting for BMI^29–32^, but this relationship had not been clearly demonstrated. In line with our results, the EPIC-Norfolk cohort reported that the risk of CAD decreased with increasing HC (adjusted for BMI) in a Cox regression model adjusted for other risk factors^33^. The association of BMI-adjusted WC with the risk of CAD from our study was in contrasted with some observational studies that have shown a positive association^17–34^. Although conditional measures of adiposity (e.g., BMI-adjusted WC) are likely to become increasingly adopted in studies, a potential problem introduced by adjusting for BMI is that the IVs may be related to a lower level of adiposity^35,36^. This can potentially lead to the MR findings being confounded. Since BMI in general increases the risk of CAD under investigation, conditional measures of adiposity will tend to bias the results to the null. For example, a prospective cohort study reported a linear association of WC with CAD, with an age-adjusted and centre-adjusted RR of 1.57 (95% CI 1.22–2.01), the RR was reduced to 0.99 (95% CI 0.76–1.30) after further adjustment for mediating and confounding factors^37^. In our study, the causal estimates of the effects of BMI-adjusted HC and WC on the risk of CAD were reversed, which requires further investigation. On the other hand, the SNPs identified to date can only explain a small percentage of the genetic variance of these traits, so there it is possible that our null results may be a consequence of limited study power if the true effect is marginal. This suggests that care is required when interpreting these results.

Our study had several strengths. First, more obesity-related traits were investigated to test associations with the risk of CAD than in previous MR studies; the extension of other measures of adiposity and fat distribution were used as exposure proxies. Second, we applied a two-sample MR approach via MR-Base, MR estimates were less biased, and any bias is in the direction of the null. Third, compared with previous MR studies, we identified SNPs for obesity-related traits as IVs from the GWASs of only individuals of European descent (controlling for population stratification), but there remained strong IVs for exposures of interest, validating assumption 1 in Fig. 1. Although the results from the MR-Egger test showed limited evidence for unbalanced horizontal pleiotropy influencing the associations, it is still possible that the relationships represent a common genetic basis rather than a causal effect due to the potential pleiotropic effects of the IVs^38^. Moreover, the power of the small percentage of the genetic variance of traits explained by the IVs is limited. Furthermore, there were at most 2.1% of individuals overlapping between the GIANT and CARDIoGRAM datasets, and the details of the results are shown in Supplementary Tables S8 and S9. As suggested in the study by Stephen Burgess and coworkers^39^, we used the F statistic to investigate the magnitude of bias arising from sample overlap based on a web application (https://sb452.shinyapps.io/overlap). The F statistic results indicated that the F parameters for obesity-related traits were large, bias would not be substantial due to sample overlap in our study. Lastly, as we only used summary statistics and had no access to the original individual clinical outcome measures, we could not conduct analyses stratified by age, sex, smoking status, or subtypes of CAD, nor could we explore the non-linearity of relationships between obesity-related traits and CAD, as had been revealed by previous studies^40,41^.

In summary, we found positive associations of BMI, HC, WC, and WHR with the risk of CAD. However, the associations of BMI-adjusted HC and WC with CAD were reversed, unlike that of WHR. Additionally, central adiposity (measured by WHR), when separated from general adiposity (measured by BMI), might pose a similar risk for CAD as general adiposity. Our findings suggested that the impact of adiposity on the risk of CAD is determined by the degree to which fat accumulates, as well as its location. In clinical practice, physicians should pay attention to the potential effects of different obesity-related traits on the risk of CAD.

## Methods and Materials

### MR Model

In the MR model, allelic variants (G), a modifiable exposure (X), and an outcome (Y) were investigated. Confounders were often unmeasured, thereby allowing for the possibility that the associations found in observational studies could not conclusively demonstrate that adiposity traits *per se* affect CAD risk. The exposure and outcome were taken as a linear function of allelic variants, and variants were assumed to provide additive contributions and took the values 0, 1 or 2 (representing the number of exposure-increasing alleles of an allelic variant). Importantly, the MR model has 3 key assumptions that must be satisfied (Fig. 1): Assumption 1: The variant is associated with the exposure (X) (e.g., obesity-related traits). Assumption 2: The variant is independent of confounders. Assumption 3: The variant influences the risk of the outcome (Y) (e.g., CAD) through the exposure and not through any other independent pathway. For each variant *j* (*j* = 1,…, j), the coefficient γ_*j*_ represents the association of the variant with the risk of the exposure, and α_*j*_ represents the association of the variant with the risk of the outcome, which is not mediated by the exposure or other potential confounders. The coefficient β is the causal effect of the exposure on the outcome and is equal to the direct association of each variant with the outcome (α) divided by the association of each variant with the exposure (γ).

### Data Sources

GWASs have identified SNPs at multiple independent loci significantly associated with obesity-related traits, including BMI, HC, WC, and WHR^42,43^. Here, we sought to use SNPs associated with obesity-related traits as IVs. Briefly, we identified SNPs associated with BMI (n = 322,154)^42^ and those associated with HC, WC, and WHR (n = 210,088)^43^ from the largest published meta-analysis of GWASs by the Genetic Investigation of Anthropometric Traits (GIANT) Consortium. Estimates of the effects of these IVs on the risk of CAD were taken from a meta-analysis of 22 GWASs by the transatlantic Coronary ARtery Disease Genome-wide Replication and Meta-analysis (CARDIoGRAM) Consortium (n = 86,995)^44^. Detailed explanations of cohort-specific characteristics are included in the Supplementary Material. Informed consent was obtained from all participants in the contributing GWASs. All contributing studies received ethical approval from their respective institutional review boards. This study was approved by the Ethical Committee of the Life Sciences of Wuhan University.

### Statistical analyses

Two-sample MR analyses were conducted using the TwoSampleMR package^45^ (available from https://github.com/MRCIEU/TwoSampleMR), which can collate and harmonize summary-level data from publicly available GWAS datasets and enable the automation and efficient implementation of our two-sample MR methodology. We used the TwoSampleMR package in R version 3.4.1 to clump and harmonize the summary-level data from the meta-analyses of GWASs for obesity-related traits (Supplementary Fig. 12). Only SNPs identified in European-ancestry GWASs were included for analysis. The proportion of variance in obesity-related traits explained by IVs in the summarized data was calculated using the grs.summary function from the gtx package in R version 3.4.1. To assess the heterogeneity of overall IVs, we derived p-values from the Q statistic.

Three tests were used for causal estimation of the association of each obesity-related trait with CAD, including the inverse-variance weighted (IVW) method, MR-Egger regression and the weighted median method. The IVW method was used to provide a combined estimate of the causal estimates from each SNP, pooling the association of each SNP with CAD using fixed-effects meta-analysis^46^. To account for potential horizontal pleiotropy, we estimated the associations using MR-Egger regression^47^. Using the MR-Egger method, each SNP’s effect on the exposure of an obesity-related trait was plotted against its effect on CAD, with the slope representing the effect estimate, and an intercept distinct from the origin provided evidence for pleiotropic effects^47^. The weighted median was applied, as it can provide valid causal estimates in the presence of horizontal pleiotropy, provided at least half of the weighted variance is valid^25^.

### Sensitivity Analysis

MR leave-one-out sensitivity analysis was performed to ascertain if each association was disproportionately influenced by a single SNP. In the MR leave-one-out sensitivity analysis, each estimate represents the IVW method-derived estimates of the effect of the exposure on the outcome, excluding that specific SNP. The overall analysis including all SNPs is also shown for comparison.

### Power Calculations

The power to detect the causal estimates was calculated using an online tool^48,49^ at http://cnsgenomics.com/shiny/mRnd/. For the causal estimate of the effect of obesity-related traits on CAD, with an available total sample size of 210,088 subjects, we had 100% statistical power to detect differences in CAD odds higher than 1.2 in the causal effect of obesity-related traits on CAD (type I error rate set at 0.05).

## Electronic supplementary material

Supplementary Material and Figures

Supplementary Tables
