# Supplementary material for: Assessing causal estimates of the association of obesity-related traits with coronary artery disease using a Mendelian randomization approach
Source: Sci Rep. 2018 May 8;8:7146. doi: 10.1038/s41598-018-25305-y (PMC5940685; doi:10.1038/s41598-018-25305-y)
Supplement: Supplementary file 1 — Supplementary Material and Figures [file 41598_2018_25305_MOESM1_ESM.doc]

Assessing causal estimates of the association of obesity-related traits with coronary artery disease using a Mendelian randomization approach

Xue Zhang^1*^, Wan-Qiang Lv^2*^, Bo Qiu^3^, Li-Jun Zhang^1^, Jian Qin^4^, Feng-Juan Tang^1^, Hai-Tao Wang^3^, Hua-Jie Li^3^, Ya-Rong Hao^1*^

^1^ Department of Geriatrics, Renmin Hospital of Wuhan University, Wuhan 430060, Hubei Province, P.R.C

^2^ Department of Epidemiology, College of Public Health, Zhengzhou University, Zhengzhou, NO.100 Kexue Road, High-Tech Development Zone Of States, P.R.C

^3^ Department of Orthopaedics, Renmin Hospital of Wuhan University, Wuhan 430060, Hubei Province, P.R.C

^4^ Central Laboratory, Renmin Hospital of Wuhan University, Wuhan 430060, Hubei Province, P.R.C

*These authors contributed equally to this work

***Corresponding author**

Ya-Rong Hao, Ph.D.

Department of Geriatrics, Renmin Hospital of Wuhan University

Wuhan 430060, Hubei Province, P.R.C

Email: [984022801@qq.com](mailto:984022801@qq.com)

**Supplementary Material**

Cohort-specific characteristics for each trait from the most recent large-scale GWAS meta-analyses were as follows:

**Body mass index**

Body mass index (BMI) summary-level GWAS data was mined from the Genetic Investigation of ANthropometric Traits (GIANT) consortium[^1^](#_ENREF_1). This consortium collected data from 339,224 individuals from 125 studies, 82 with GWAS results and 43 with results from Metabochip. Following study-specific quality control measures, all contributing GWAS common genetic variants were imputed using the HapMap phase II CEU reference panel for European-descent studies, and HapMap release 22 for the African-American and Hispanic GWAS. Directly genotyped (GWAS and Metabochip) and imputed variants (GWAS only) were then tested for association with the inverse normally transformed BMI residuals using linear regression assuming an additive genetic model. Quality control following study level analyses was conducted following procedures outlined elsewhere[^2^](#_ENREF_2).

**Waist-hip ratio adjusted for BMI, hip circumference adjusted for BMI, and waist circumference adjusted for BMI**

These GWAS datasets were mined from the Genetic Investigation of ANthropometric Traits (GIANT) consortium[^3^](#_ENREF_3). In our study, we only used the meta-analysis of GWASs from European ancestry. The European ancestry arm included 142,762 individuals from 57 cohorts genotyped with genome-wide SNP arrays and 67,326 individuals from 44 cohorts genotyped with the Metabochip11. There was no overlap between individuals genotyped with genome-wide SNP arrays and Metabochip. Sample and SNP quality control (QC) were undertaken within each cohort. Association summary statistics from the two parts of the European ancestry arm were combined via inverse-variance weighted fixed-effects meta-analysis using METAL with no further genomic control correction. Results were reported for SNPs with a sex-combined sample size≥50,000. Analyses were corrected for population structure within each sex.

**Coronary artery disease**

Coronary artery disease (CAD) summary-level GWAS data was downloaded from the transatlantic Coronary ARtery DIsease Genome wide Replication and Meta-analysis (CARDIoGRAM) consortium[^4^](#_ENREF_4). This study included a meta-analysis of 14 GWASs of CAD, comprising 22,233 cases and 64,762 controls, all of European ancestry. Additionally, they genotyped lead SNPs within the most promising novel loci as well as a subset of previously reported CAD loci in up to 60,738 additional subjects. Quality control following study level analyses was conducted following procedures outlined elsewhere. All contributing GWAS common genetic variants were imputed using the HapMap phase II CEU reference panel for European-descent studies.

**References**

1 Locke, A. E. et al. Genetic studies of body mass index yield new insights for obesity biology. *Nature* **518**, 197-206 (2015).

2 Winkler, T. W. et al. Quality control and conduct of genome-wide association meta-analyses. *Nat Protoc* **9**, 1192-1212 (2014).

3 Shungin, D. et al. New genetic loci link adipose and insulin biology to body fat distribution. *Nature* **518**, 187-196 (2015).

4 Mehta, N. N. Large-scale association analysis identifies 13 new susceptibility loci for coronary artery disease. *Circ Cardiovasc Genet* **4**, 327-329 (2011).

**Supplementary Figures**

**Supplementary Fig. 1**: MR forest plot for body mass index on risk of coronary artery disease.

**Supplementary Fig. 2**: MR funnel plot for body mass index on risk of coronary artery disease.

**Supplementary Fig. 3**: MR leave-one-out sensitivity analysis for body mass index on risk of coronary artery disease.

**Supplementary Fig. 4**: MR leave-one-out sensitivity analysis for hip circumference on risk of coronary artery disease.

**Supplementary Fig. 5**: MR leave-one-out sensitivity analysis for hip circumference adjusted for BMI on risk of coronary artery disease.

**Supplementary Fig. 6**: MR leave-one-out sensitivity analysis for waist circumference on risk of coronary artery disease.

**Supplementary Fig. 7**: MR leave-one-out sensitivity analysis for waist circumference adjusted for BMI on risk of coronary artery disease.

**Supplementary Fig. 8**: MR leave-one-out sensitivity analysis for waist-hip ratio on risk of coronary artery disease.

**Supplementary Fig. 9**: MR leave-one-out sensitivity analysis for waist-hip ratio adjusted for BMI on risk of coronary artery disease.

**Supplementary Fig. 10**: Illustration of independent and overlap SNPs between any two traits of BMI, HC, WC and WHR.

**Supplementary Fig. 11**: Illustration of independent and overlap SNPs between any two traits of BMI and BMI-adjusted HC, WC, WHR.

**Supplementary Fig. 12:** Illustration of selecting SNPs for assessing the association of the exposure (obesity-related traits) with the outcome (CAD).


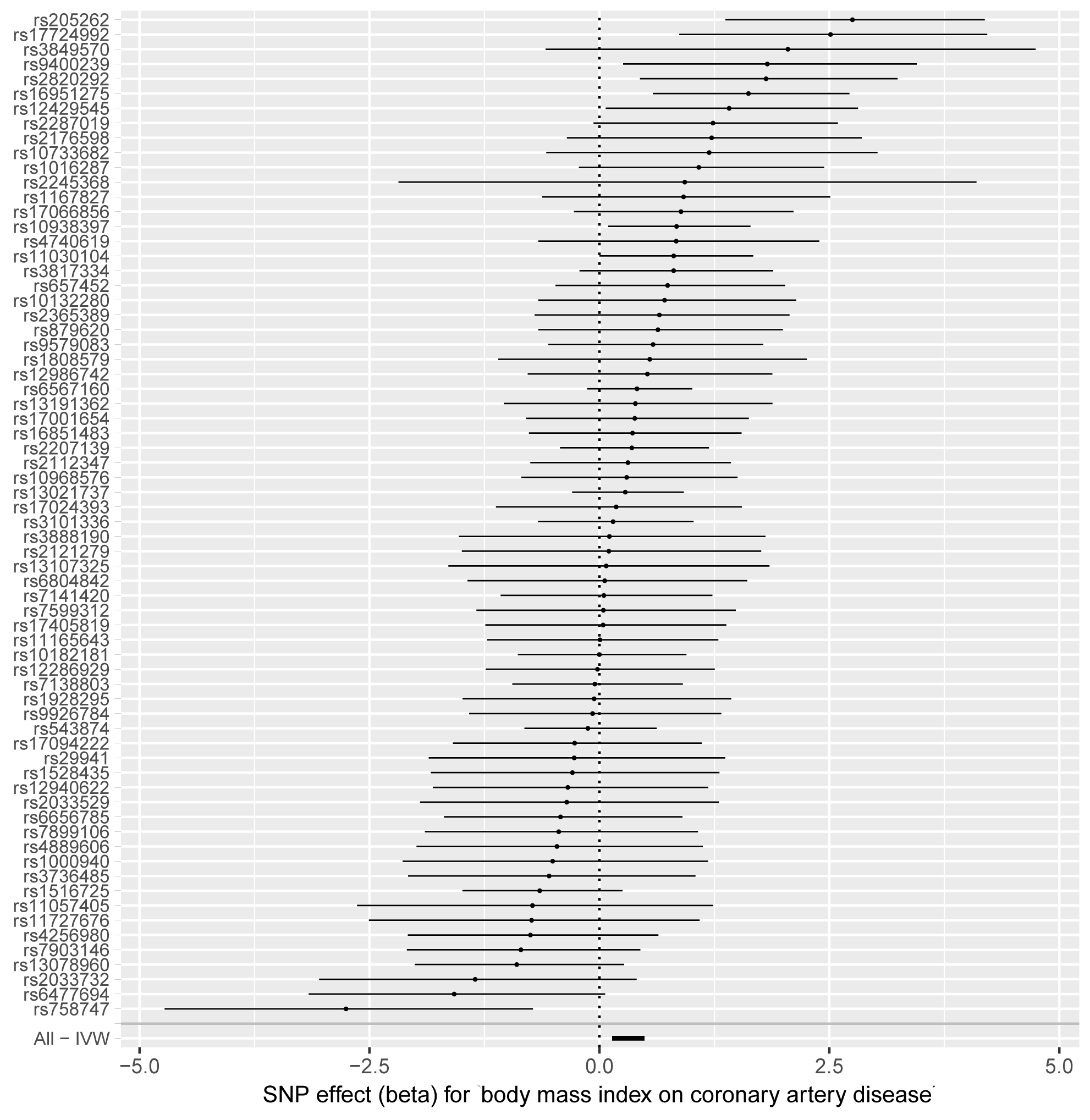


**Supplementary Fig. 1**: MR forest plot for body mass index on risk of coronary artery disease. For each of the 68 BMI SNPs, the forest plot show the IVW-derived effect estimate of BMI on coronary artery disease. The overall analysis including all SNPs is also shown for comparison.


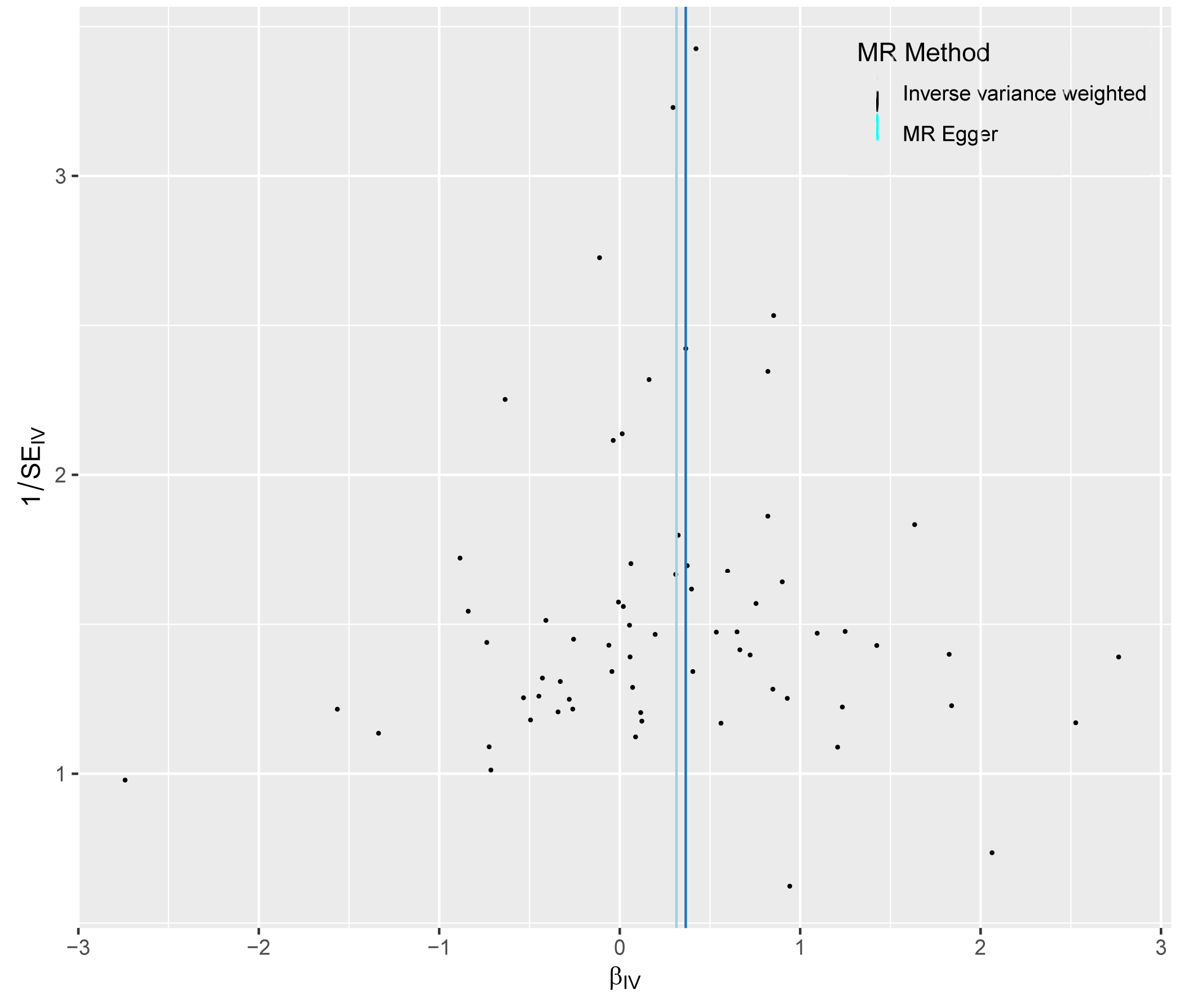


**Supplementary Fig. 2**: MR funnel plot for body mass index on risk of coronary artery disease. Funnel plot showing the relationship between the causal effect of exposure on outcome estimated by each SNP against the inverse of the standard error of the causal estimate. Vertical lines show the MR estimates using all SNPs.


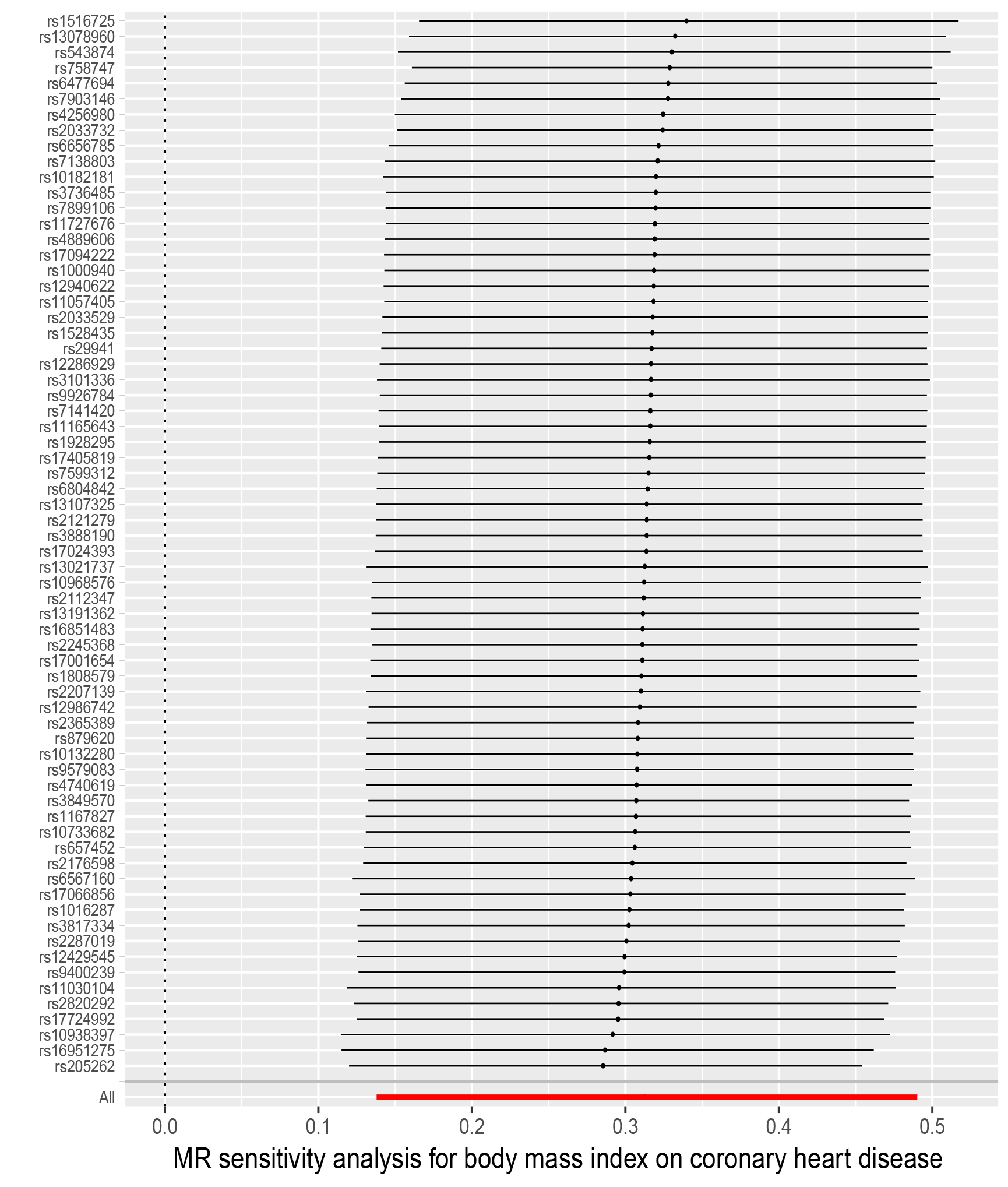


**Supplementary Fig. 3**: MR leave-one-out sensitivity analysis for body mass index on risk of coronary artery disease. Each estimate represents the IVW-derived effect estimates of the exposure on outcome, excluding that particular SNP. The overall analysis including all SNPs is also shown for comparison.


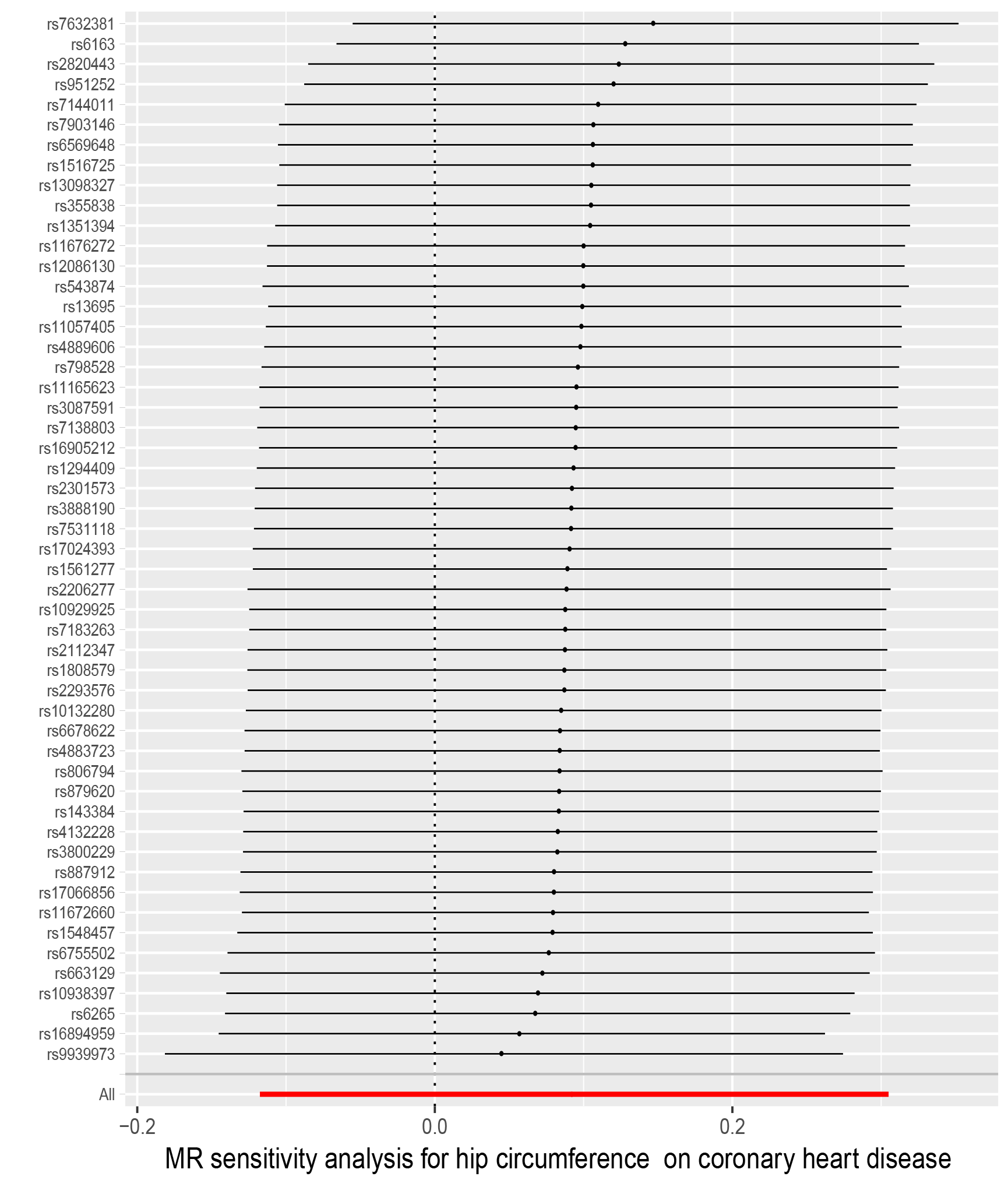


**Supplementary Fig. 4**: MR leave-one-out sensitivity analysis for hip circumference on risk of coronary artery disease.


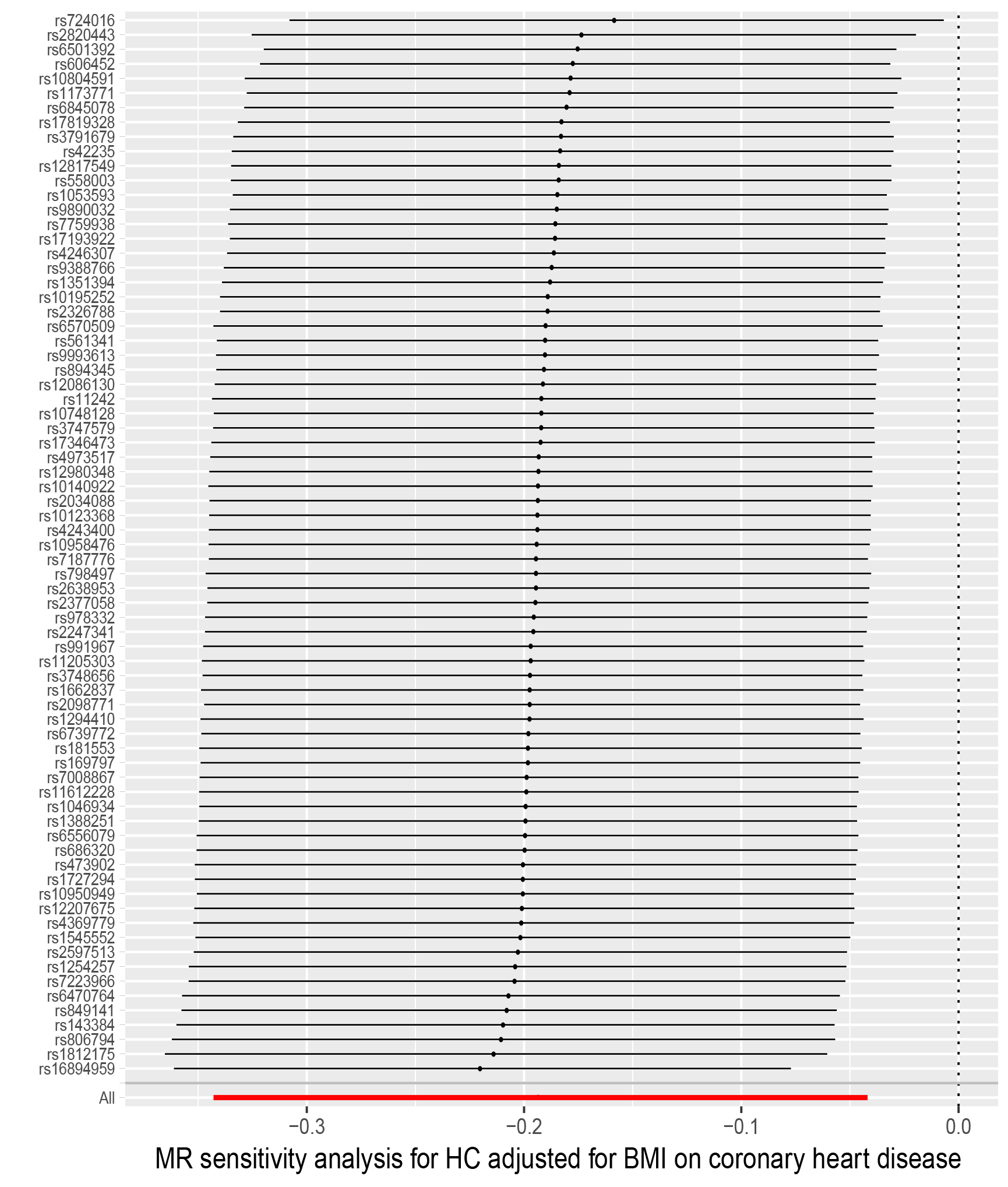


**Supplementary Fig. 5**: MR leave-one-out sensitivity analysis for hip circumference adjusted for BMI on risk of coronary artery disease.


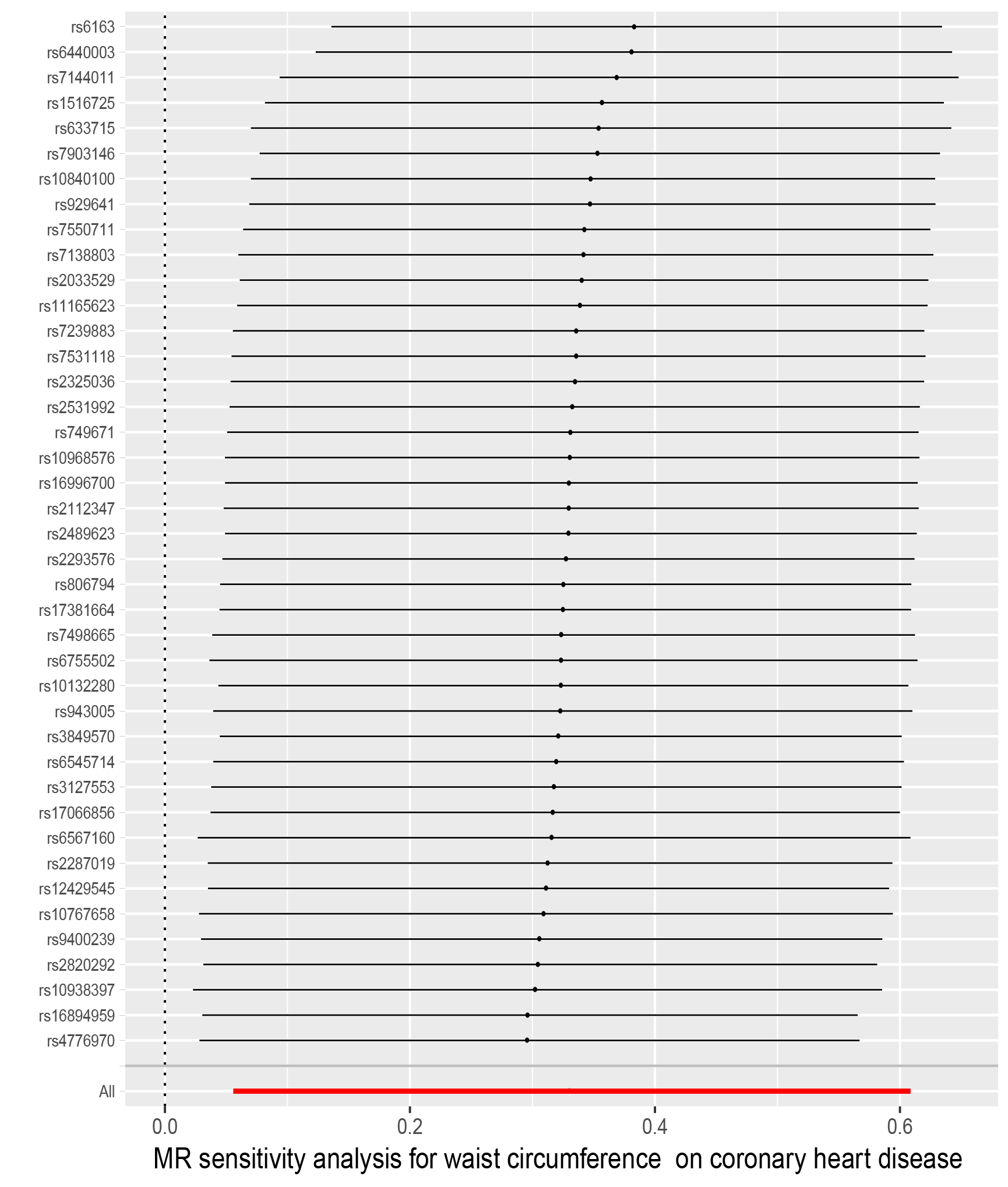


**Supplementary Fig. 6**: MR leave-one-out sensitivity analysis for waist circumference on risk of coronary artery disease.
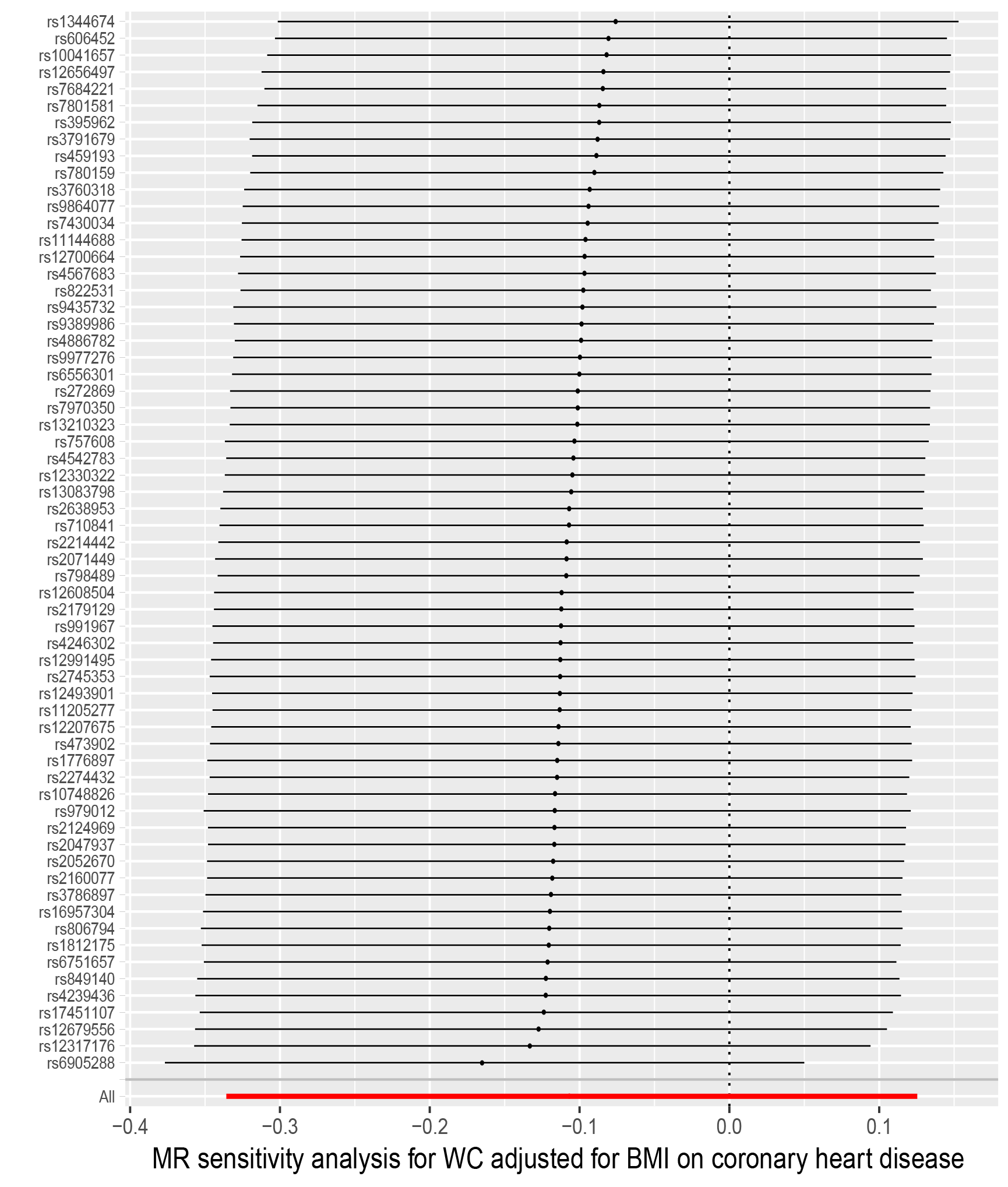


**Supplementary Fig. 7**: MR leave-one-out sensitivity analysis for waist circumference adjusted for BMI on risk of coronary artery disease.
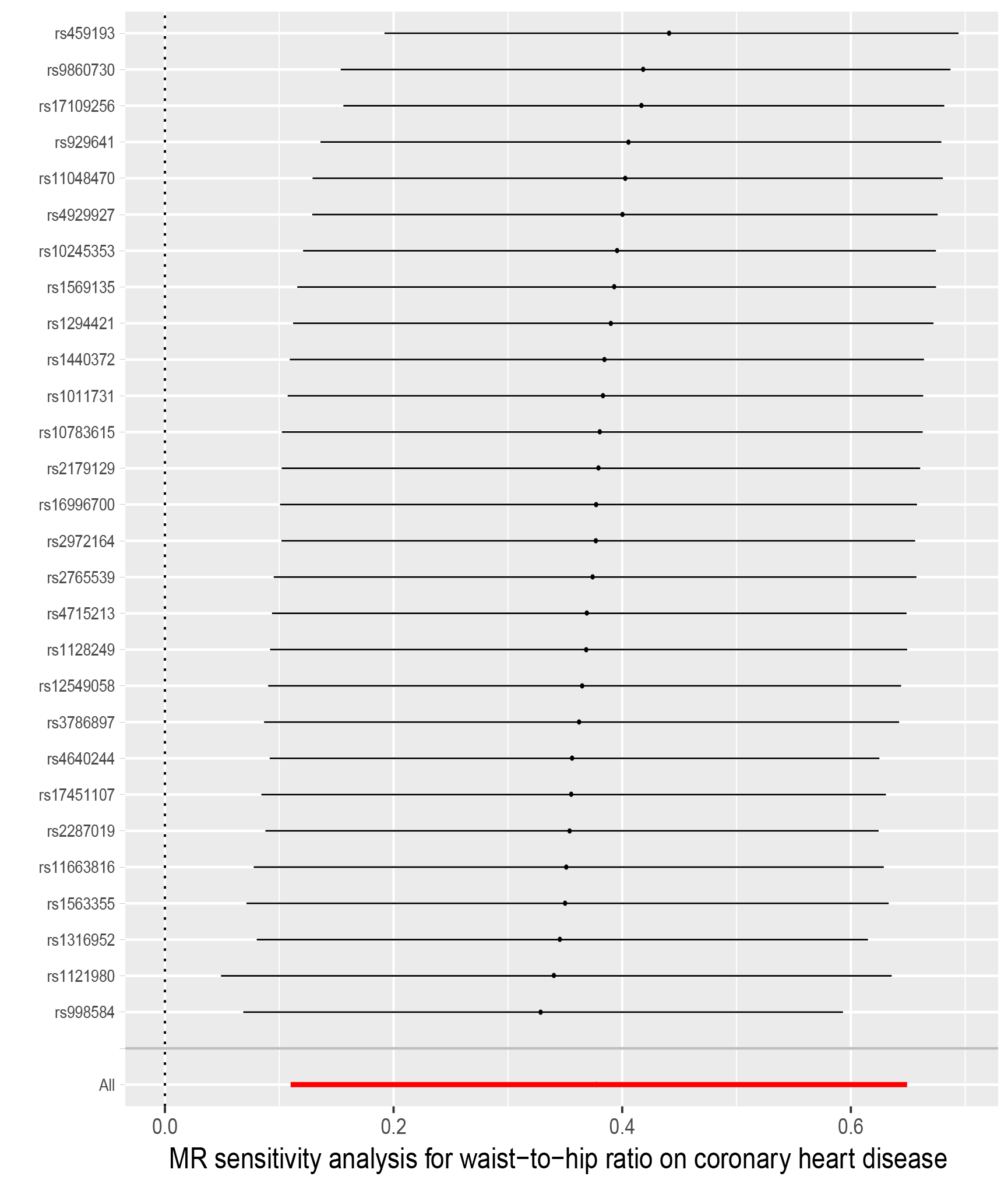
 **Supplementary Fig. 8**: MR leave-one-out sensitivity analysis for waist-hip ratio on risk of coronary artery disease.


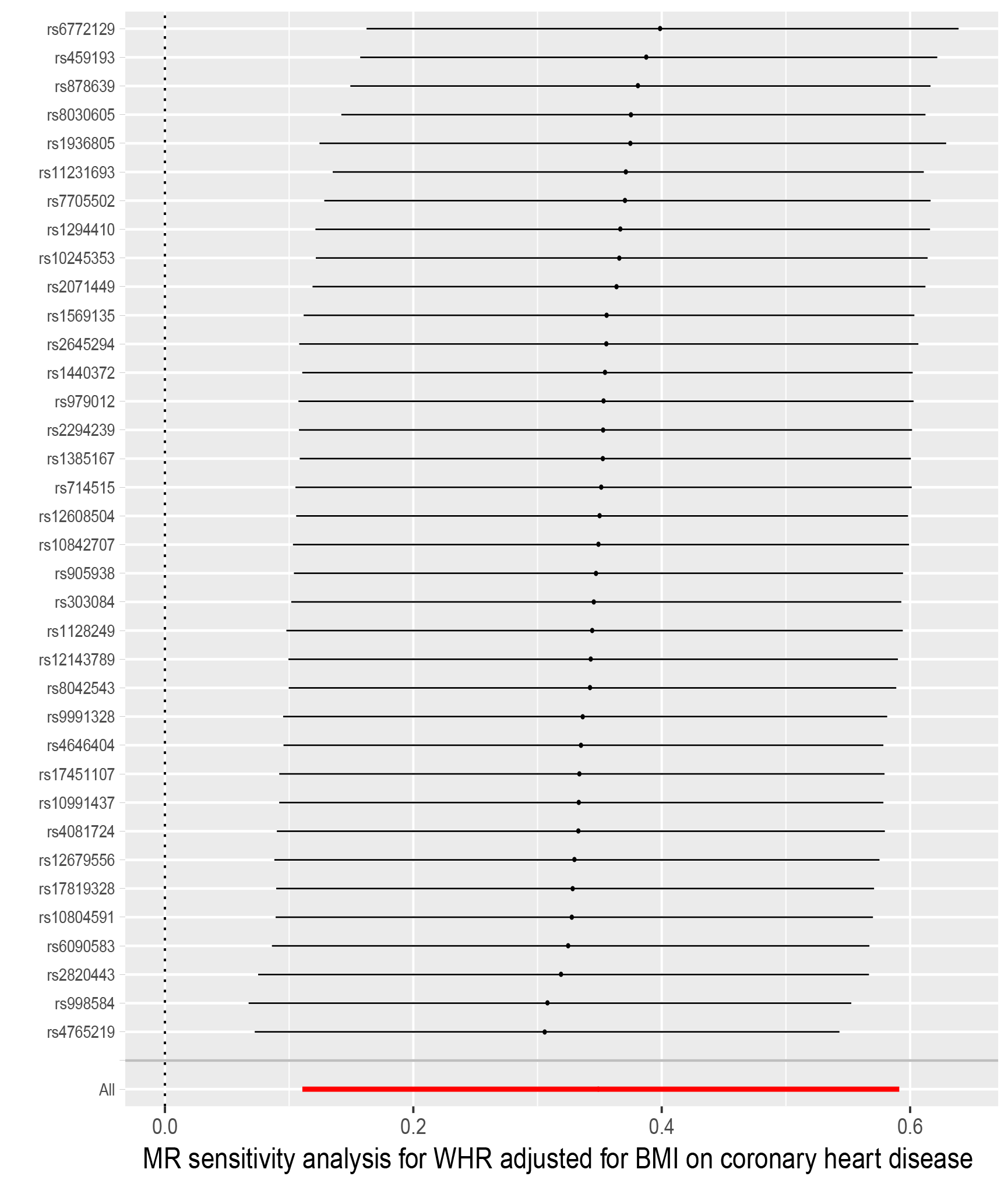


**Supplementary Fig. 9**: MR leave-one-out sensitivity analysis for waist-hip ratio adjusted for BMI on risk of coronary artery disease.


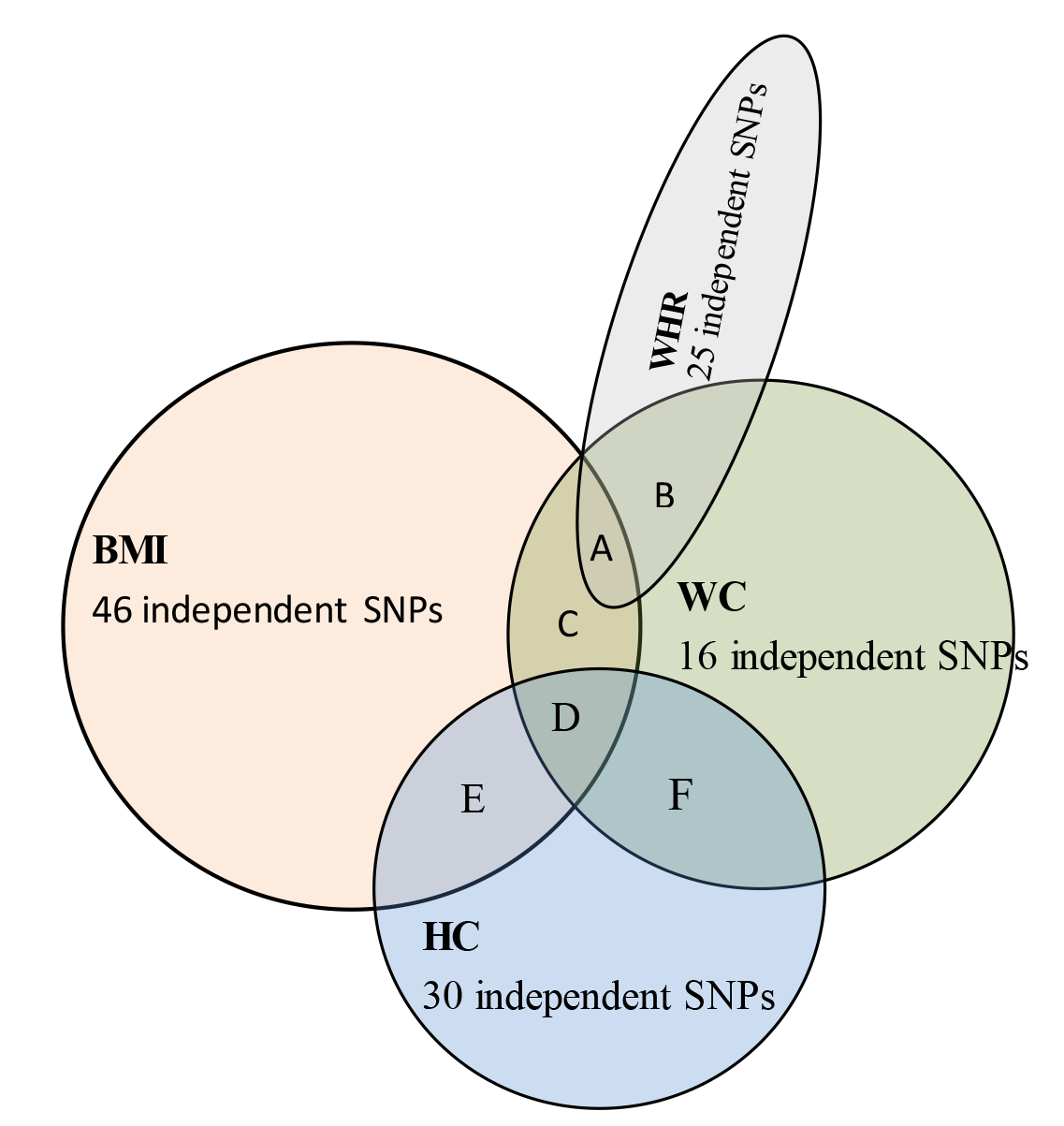


**Supplementary Fig. 10:** Illustration of independent and overlap SNPs between any two traits of BMI, HC, WC and WHR. Section A represents the overlap between BMI and WHR SNPs, including one SNP (rs2287019); section B represents the overlap between WC and WHR SNPs except for section A, including 2 SNPs (rs16996700, rs929641); section C represents the overlap between BMI and WC SNPs except for section A and D, including 7 SNPs (rs10968576, rs12429545, rs2033529, rs2820292, rs3849570, rs6567160, rs9400239); section D represents the overlap between BMI, HC and WC SNPs, including 7 SNPs (rs10132280, rs10938397, rs1516725, rs17066856, rs2112347, rs7138803, rs7903146); section E represents the overlap between BMI and HC SNPs except for section D, including 7 SNPs (rs11057405, rs17024393, rs1808579, rs3888190, rs4889606, rs543874, rs879620); section F represents the overlap between HC and WC SNPs except for section D, including 8 SNPs (rs11165623, rs16894959, rs2293576, rs6163, rs6755502, rs7144011, rs7531118, rs806794).


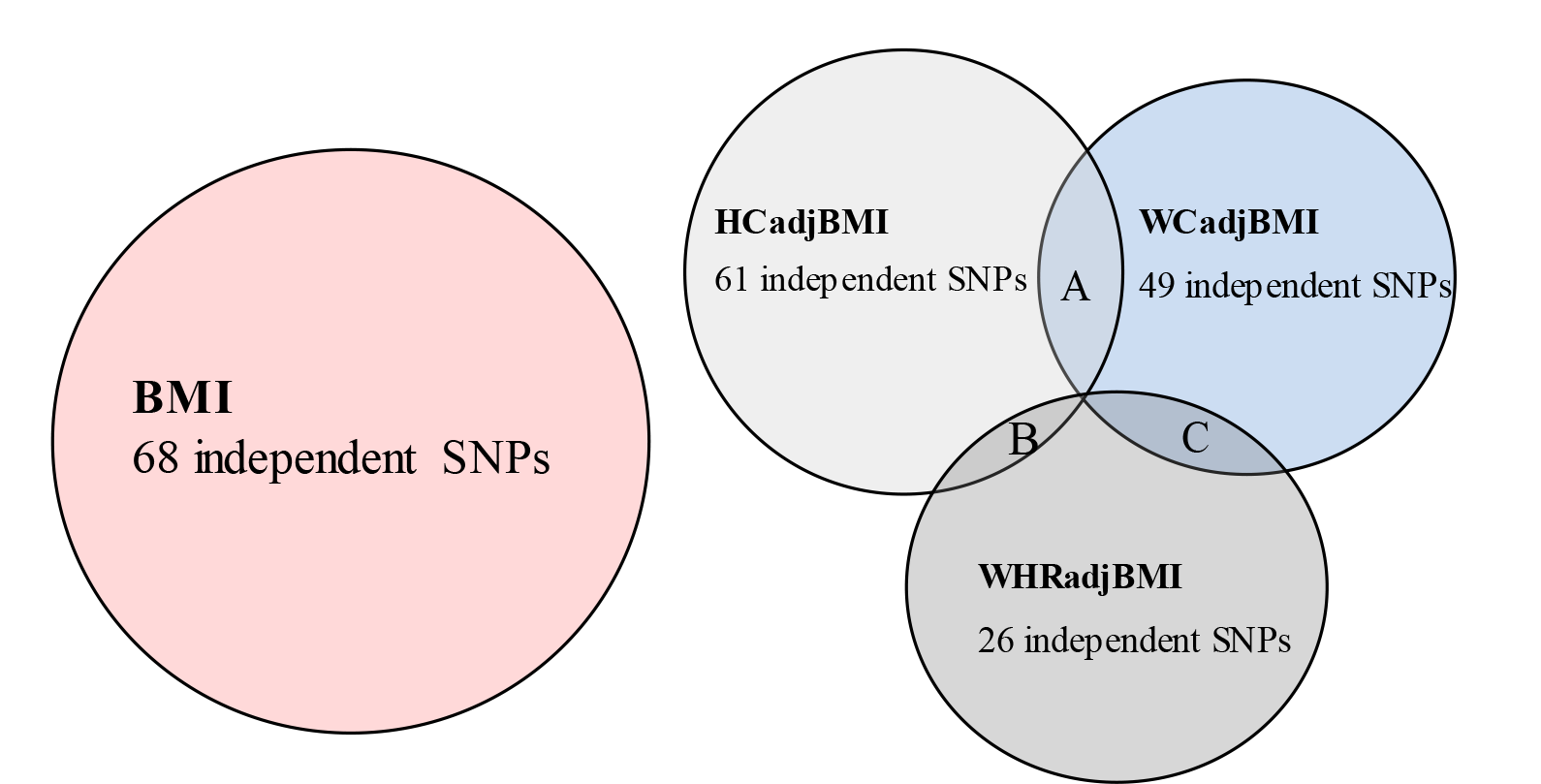


**Supplementary Fig. 11:** Illustration of independent and overlap SNPs between any two traits of BMI and BMI-adjusted HC, WC and WHR. Section A represents the overlap between HCadjBMI and WCadjBMI SNPs, including 8 SNPs (rs12207675, rs1812175, rs2638953, rs3791679, rs473902, rs606452, rs806794, rs991967); section B represents the overlap between HCadjBMI and WHRadjBMI SNPs, including 4 SNPs (rs10804591, rs1294410, rs17819328, rs2820443); section C represents the overlap between WCadjBMI and WHRadjBMI SNPs, including 6 SNPs (rs12608504, rs12679556, rs17451107, rs2071449, rs459193, rs979012).

**
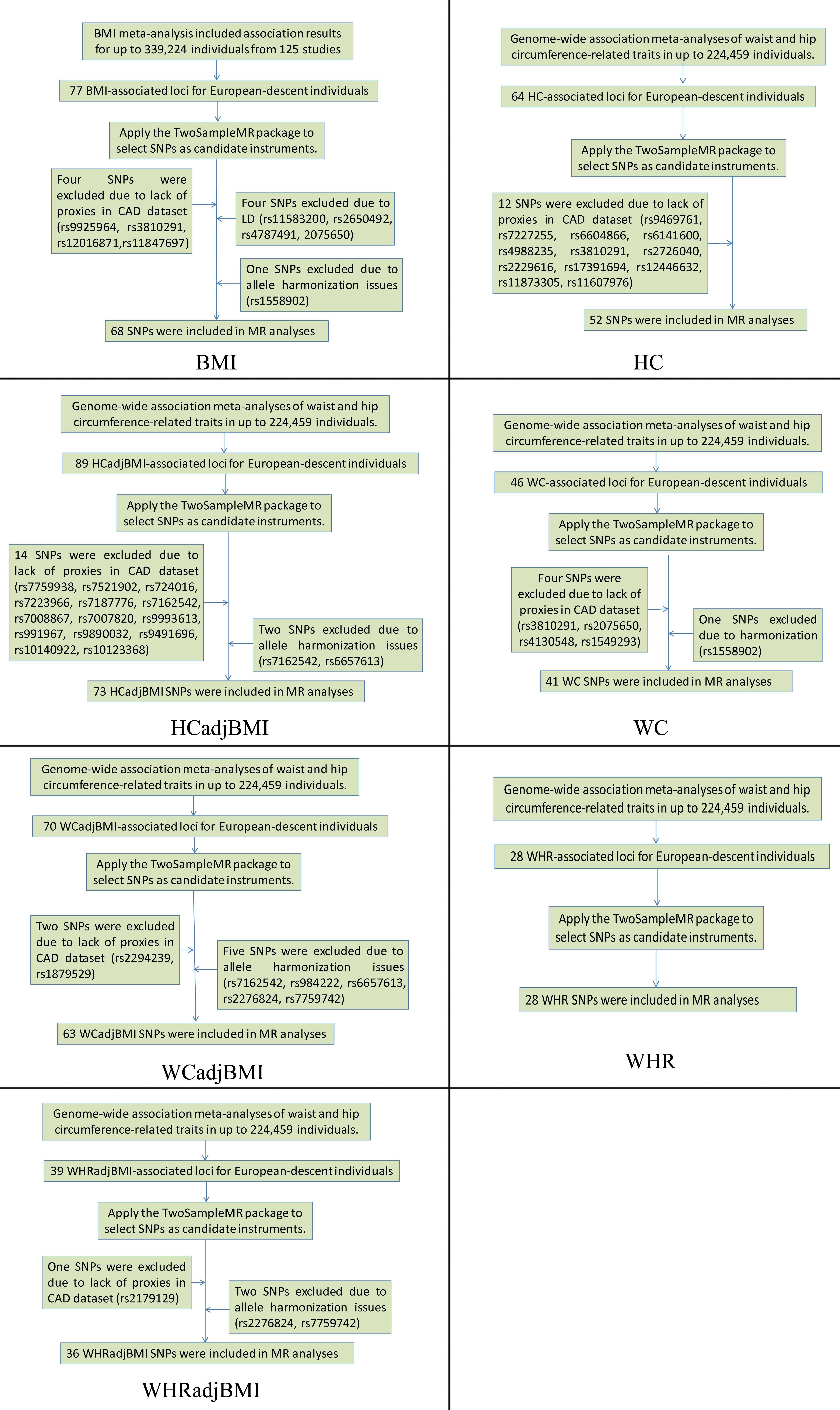
**

**Supplementary Fig. 12:** Illustration of selecting SNPs for assessing the association of the exposure (obesity-related traits) with the outcome (CAD).
